# Correction: A novel TRPV5/6-like channel from a scleractinian coral

**DOI:** 10.1371/journal.pone.0340231

**Published:** 2026-01-02

**Authors:** The PLOS ONE Staff

## Notice of Republication

This article was republished on December 18, 2025, to correct errors in the Supporting Information files. Incorrect versions of S2 Fig, S3 Fig, and S4 Fig were published in error, and the correct S4 Fig file was missing entirely. The publisher apologises for these errors. Please download this article again to view the correct version.

## Supporting information

S1 File Originally published, uncorrected article. (PDF)

S2 File Republished, corrected article. (PDF)

S2 Fig Sequence logo visualization for the pore and TRP box in TRPV5, TRPV6 and ancestor-like channels. (A) Consensus amino acid sequences of the pore domain (selectivity filter, central cavity, and gate) for the indicated groups of organisms. The colored residues are the most conserved or demonstrated to have important functional roles in channel behavior. (B) Consensus amino acid sequences for the TRP box domain depicting highly conserved residues across mammals, sauropsids, fish, arthropods, mollusks, and cnidarians.
(TIF)

S3 Fig HEK293 cells lack endogenous TRPV5 channel expression. (A) Representative whole-cell current traces from untransfected HEK293 cells and cells transfected with either human TRPV5 or coral PdTRPV5/6-like channels. (B) The corresponding whole-cell current–voltage (I–V) relationships are shown, with error bars representing the standard error of the mean (± s.e.m.).
(TIF)

S4 Fig Effect of intracellular ATP-diNa in PdTRPV5/6-like and hTRPV5 channels. (A and C) Time course (5-min with 3s intervals) of monovalent currents recorded at −100 mV after patch excision in the outside-out configuration (light blue and orange circles) in the absence of ATP. The inward currents increased over time in the presence of 10mM ATP-diNa (n = 5) (dark blue and red circles) for both species, respectively. (B and D) Representative current traces with prolonged exposure to ATP-diNa, which prevents rundown of TRPV5 channels in symmetrical conditions of monovalent cations. The dotted line indicates the final current after 5 minutes. Normalized currents show the average of each experiment with error bars for both conditions (shadows). Data are mean ± s.e.m.
(TIF)
