# Supplementary material for: A novel TRPV5/6-like channel from a scleractinian coral
Source: PLoS One. 2025 Oct 7;20(10):e0332671. doi: 10.1371/journal.pone.0332671 (PMC12503276; doi:10.1371/journal.pone.0332671)
Supplement: S1 Table — List of TRPV5 and TRPV6 protein sequences retrieved from NCBI grouped by phylum. The table includes the species name and corresponding accession number for each sequence. (DOCX) [file pone.0332671.s001.docx]

| **Supplementary Table 1**. Accession numbers of the TRPV5 and TRPV6 sequences used in this study   \| >XP_027049851.PdTRPV5/6-like/372-649 Pocillopora damicornis CNIDARIO-1 \| \| --- \| \| >NP_001076126.1/310-609 TRPV5 Oryctolagus cuniculus MAMMAL-1 \| \| >AAY34564.1/310-609 TRPV6 Oryctolagus cuniculus MAMMAL-2 \| \| >RDD41986.1/404-711 TRPV6 Trichoplax sp. H2 PLACOZOA-1 \| \| >RDD40584.1/386-664 TRPV6 Trichoplax sp. H2 PLACOZOA-2 \| \| >XP_062515099.1/395-673 TRPV5-like Corticium candelabrum PORIFERA-1 \| \| >XP_062518232.1/160-476 TRPV6-like Corticium candelabrum PORIFERA-2 \| \| >XP_049749856.1/311-610 TRPV5 Elephas maximus indicus MAMMAL-3 \| \| >XP_058387887.1/310-609 TRPV5 Diceros bicornis minor MAMMAL-4 \| \| >NP_001157428.1/310-609 TRPV5 Equus caballus MAMMAL-5 \| \| >XP_058387877.1/310-609 TRPV6 Diceros bicornis minor MAMMAL-6 \| \| >XP_001490955.2/310-609 TRPV6 Equus caballus MAMMAL-7 \| \| >NP_062815.3/310-609 TRPV5 Homo sapiens MAMMAL-8 \| \| >NP_061116.5/350-649 TRPV6 Homo sapiens MAMMAL-9 \| \| >NXE56193.1/312-611 TRPV6 Casuarius casuarius BIRD-1 \| \| >NXA55958.1/312-611 TRPV6 Nothocercus julius BIRD-2 \| \| >XP_030309629.1/312-611 TRPV6 Calypte anna BIRD-3 \| \| >XP_013056258.1/312-611 TRPV6-like Anser cygnoides BIRD-4 \| \| >NWX86897.1/312-611 TRPV5 Nothoprocta pentlandii BIRD-5 \| \| >XP_051499437.1/312-611 TRPV6-like Apus apus BIRD-6 \| \| >XP_054044082.1/312-611 TRPV5 Rissa tridactyla BIRD-7 \| \| >NXA41878.1/312-611 TRPV6 Eudromia elegans BIRD-8 \| \| >NXF54516.1/312-611 TRPV5 Oceanites oceanicus BIRD-9 \| \| >NXC41637.1/312-611 TRPV5 Penelope pileata BIRD-10 \| \| >XP_040430109.1/312-611 TRPV6-like Cygnus olor BIRD-11 \| \| >KQL60723.1/312-611 TRPV6 Amazona aestiva BIRD-12 \| \| >XP_030807303.1/312-611 TRPV6-like Camarhynchus parvulus BIRD-13 \| \| >NWI12810.1/312-611 TRPV6 Crypturellus soui BIRD-14 \| \| >XP_025900238.1/312-611 TRPV6-like Nothoprocta perdicaria BIRD-15 \| \| >NXE26995.1/312-611 TRPV5 Ardeotis kori BIRD-16 \| \| >NWZ55310.1/312-611 TRPV5 Haliaeetus albicilla BIRD-17 \| \| >NXW88055.1/312-611 TRPV6 Alopecoenas beccarii BIRD-18 \| \| >XP_049680517.1/312-611 TRPV6-like Accipiter gentilis BIRD-19 \| \| >XP_024106101.2/310-609 TRPV6 Pongo abelii MAMMAL-10 \| \| >XP_002818624.1/310-609 TRPV5 Pongo abelii MAMMAL-11 \| \| >XP_047704423.1/310-609 TRPV5 Prionailurus viverrinus MAMMAL-12 \| \| >XP_047705675.1/310-609 TRPV6 Prionailurus viverrinus MAMMAL-13 \| \| >XP_047550837.1/310-609 TRPV5 Lutra lutra MAMMAL-14 \| \| >XP_058580004.1/310-609 TRPV5 Neofelis nebulosa MAMMAL-15 \| \| >XP_044908848.1/310-609 TRPV5 Felis catus MAMMAL-16 \| \| >XP_025837968.1/310-609 TRPV5 Vulpes vulpes MAMMAL-17 \| \| >XP_040327671.1/310-609 TRPV5 Puma yagouaroundi MAMMAL-18 \| \| >XP_045351919.1/310-609 TRPV5 Leopardus geoffroyi MAMMAL-19 \| \| >XP_025838019.1/310-609 TRPV6 Vulpes vulpes MAMMAL-20 \| \| >XP_039098980.1/310-609 TRPV5 Hyaena hyaena MAMMAL-21 \| \| >XP_039098984.1/310-609 TRPV6 Hyaena hyaena MAMMAL-22 \| \| >XP_019308565.1/310-609 TRPV6 Panthera pardus MAMMAL-23 \| \| >XP_053754375.1/310-609 TRPV5 Panthera pardus MAMMAL-24 \| \| >XP_009202289.3/310-609 TRPV6 Papio anubis MAMMAL-25 \| \| >XP_022434851.1/310-609 TRPV5 Delphinapterus leucas MAMMAL-26 \| \| >XP_022434888.1/336-635 TRPV6 Delphinapterus leucas MAMMAL-27 \| \| >XP_059877014.1/310-609 TRPV6 Delphinus delphis MAMMAL-28 \| \| >XP_059876017.1/310-609 TRPV5 Delphinus delphis MAMMAL-29 \| \| >NP_001007573.1/303-602 TRPV5 Mus musculus MAMMAL-30 \| \| >XP_057360831.1/310-609 TRPV5 Manis pentadactyla MAMMAL-31 \| \| >KAI5274019.1/317-616 TRPV6 Manis pentadactyla MAMMAL-32 \| \| >NP_071858.3/349-648 TRPV6 Mus musculus MAMMAL-33 \| \| >XP_032762568.1/315-614 TRPV6 Rattus rattus MAMMAL-34 \| \| >NP_446239.3/303-602 TRPV5 Rattus norvegicus MAMMAL-35 \| \| >XP_008152192.2/350-649 TRPV6 Eptesicus fuscus MAMMAL-36 \| \| >XP_058959373.1/409-726 TRPV5-like Pocillopora verrucosa CNIDARIA-2 \| \| >XP_022783677.1/381-701 TRPV5-like Stylophora pistillata CNIDARIA-3 \| \| >XP_020623877.1/402-731 TRPV5-like Orbicella faveolata CNIDARIA-4 \| \| >XP_029197171.2/407-728 TRPV5-like Acropora millepora CNIDARIA-5 \| \| >XP_048584484.1/420-743 TRPV6 Nematostella vectensis CNIDARIA-6 \| \| >XP_022791451.1/372-649 TRPV6-like Stylophora pistillata CNIDARIA-7 \| \| >XP_033095745.1/401-690 TRPV5-like Anneissia japonica ECHINODERM-1 \| \| >XP_022081163.1/402-679 TRPV6-like Acanthaster planci ECHINODERM-2 \| \| >XP_038069039.1/401-682 TRPV5-like Patiria miniata ECHINODERM-3 \| \| >XP_033647153.1/402-696 TRPV5-like Asterias rubens ECHINODERM-4 \| \| >XP_038069043.1/402-679 TRPV6-like Patiria miniata ECHINODERM-5 \| \| >XP_030856295.1/395-675 TRPV5 Strongylocentrotus purpuratus ECHINODERM-6 \| \| >XP_041460454.1/397-677 TRPV5-like Lytechinus variegatus ECHINODERM-7 \| \| >XP_033647957.1/397-678 TRPV6-like Asterias rubens ECHINODERM-8 \| \| >XP_041460032.1/418-716 TRPV5-like Lytechinus variegatus ECHINODERM-9 \| \| >XP_054750605.1/299-597 TRPV6-like Lytechinus pictus ECHINODERM-10 \| \| >XP_022080632.1/389-694 TRPV5-like Acanthaster planci ECHINODERM-11 \| \| >XP_786430.2/384-685 TRPV5 Strongylocentrotus purpuratus ECHINODERM-12 \| \| >XP_041477410.1/384-685 TRPV5-like Lytechinus variegatus ECHINODERM-13 \| \| >XP_030841827.1/415-716 TRPV5-like Strongylocentrotus purpuratus ECHINODERM-14 \| \| >XP_033633353.1/397-698 TRPV5-like Asterias rubens ECHINODERM-15 \| \| >XP_030829283.1/415-716 TRPV6-like Strongylocentrotus purpuratus ECHINODERM-16 \| \| >XP_022080957.1/344-625 TRPV6-like Acanthaster planci ECHINODERM-17 \| \| >XP_033098366.1/306-560 TRPV5-like Anneissia japonica ECHINODERM-18 \| \| >XP_044855676.1/311-610 TRPV6-like Mauremys mutica REPTILE-1 \| \| >XP_007068670.1/311-610 TRPV6 Chelonia mydas REPTILE-2 \| \| >XP_032632312.1/311-610 TRPV6-like Chelonoidis abingdonii REPTILE-3 \| \| >XP_048677827.1/311-610 TRPV6-like Caretta caretta REPTILE-4 \| \| >XP_005281725.1/311-610 TRPV6 Chrysemys picta bellii REPTILE-5 \| \| >XP_050801175.1/311-610 TRPV6-like Gopherus flavomarginatus REPTILE-6 \| \| >XP_026516030.1/311-610 TRPV6-like Terrapene carolina triunguis REPTILE-7 \| \| >XP_029196985.2/370-647 TRPV6-like Acropora millepora CNIDARIA-8 \| \| >KAK2571192.1/352-629 TRPV6 Acropora cervicornis CNIDARIA-9 \| \| >XP_048577476.1/369-648 TRPV6 Nematostella vectensis CNIDARIA-10 \| \| >XP_031553642.1/362-640 TRPV6-like Actinia tenebrosa CNIDARIA-11 \| \| >XP_029197180.2/239-560 TRPV6-like Acropora millepora CNIDARIA-12 \| \| >XP_027039789.1/409-726 TRPV5-like Pocillopora damicornis CNIDARIA-13 \| \| >XP_031563077.1/415-735 TRPV5-like Actinia tenebrosa CNIDARIA-14 \| \| >KAG5336807.1/350-791 TRPV5 Acromyrmex heyeri ARTHROPOD-1 \| \| >XP_026467431.1/390-732 TRPV5 Ctenocephalides felis ARTHROPOD-2 \| \| >XP_023309951.1/389-726 TRPV5 Anoplophora glabripennis ARTHROPOD-3 \| \| >XP_044267903.1/389-731 TRPV5 Tribolium madens ARTHROPOD-4 \| \| >XP_055693249.1/390-754 TRPV6 Lutzomyia longipalpis ARTHROPOD-5 \| \| >XP_026764431.1/390-766 TRPV5 Galleria mellonella ARTHROPOD-6 \| \| >XP_023026437.1/389-744 TRPV5 Leptinotarsa decemlineata ARTHROPOD-7 \| \| >XP_059608807.1/390-743 TRPV5 Phlebotomus argentipes ARTHROPOD-8 \| \| >XP_002021384.1/390-749 TRPV6 Drosophila persimilis ARTHROPOD-9 \| \| >XP_017068195.2/390-748 TRPV5 Drosophila eugracilis ARTHROPOD-10 \| \| >XP_045489488.1/390-732 TRPV5 Pieris rapae ARTHROPOD-11 \| \| >XP_060516744./389-749 1TRPV5 Cylas formicarius ARTHROPOD-12 \| \| >XP_037880283.1/390-763 TRPV5 Glossina fuscipes ARTHROPOD-14 \| \| >XP_048520785.1/389-746 TRPV5 Dendroctonus ponderosae ARTHROPOD-15 \| \| >XP_017298846.1/388-733 TRPV5 Diaphorina citri ARTHROPOD-16 \| \| >XP_046993972.1/386-721 TRPV5 Schistocerca americana ARTHROPOD-17 \| \| >XP_045472596.1/389-737 TRPV5 Harmonia axyridis ARTHROPOD-18 \| \| >XP_029405289.2/390-761 TRPV5 Bactrocera dorsalis ARTHROPOD-19 \| \| >XP_037068834.1/364-646 TRPV6-like Pollicipes pollicipes ARTHROPOD-20 \| \| >KYN43668.1/350-791 TRPV6 Trachymyrmex septentrionalis ARTHROPOD-21 \| \| >XP_026301798.1/372-657 TRPV5 Apis mellifera ARTHROPOD-22 \| \| >XP_048524004.1/375-677 TRPV6 Dendroctonus ponderosae ARTHROPOD-23 \| \| >XP_049784355.1/375-659 TRPV5 Schistocerca cancellata ARTHROPOD-24 \| \| >XP_050576738.1/372-657 TRPV5 Bombus affinis ARTHROPOD-25 \| \| >XP_023290648.1/371-656 TRPV5 Orussus abietinus ARTHROPOD-26 \| \| >XP_024215127.1/293-575 TRPV6 Halyomorpha halys ARTHROPOD-27 \| \| >XP_005173525.1/308-608 TRPV6 Danio rerio FISH-1 \| \| >XP_061091779.1/312-612 TRPV6 Conger conger FISH-2 \| \| >XP_048117834.1/312-612 TRPV6 Alosa alosa FISH-3 \| \| >XP_030639337.1/312-612 TRPV5-like Chanos chanos FISH-4 \| \| >XP_046887751.1/312-612 TRPV6 Hypomesus transpacificus FISH-5 \| \| >XP_029359829.1/312-612 TRPV5-like Echeneis naucrates FISH-6 \| \| >XP_022612418.1/312-612 TRPV6-like Seriola dumerili FISH-7 \| \| >XP_026173248.1/312-612 TRPV5 Mastacembelus armatus FISH-8 \| \| >CAJ1062458.1/312-612 TRPV6 Xyrichtys novacula FISH-9 \| \| >XP_004078007.1/312-612 TRPV5 Oryzias latipes FISH-10 \| \| >XP_035018518.1/312-612 TRPV6 Hippoglossus stenolepis FISH-11 \| \| >XP_031708721.1/312-612 TRPV5 Anarrhichthys ocellatus FISH-12 \| \| >XP_029928863.1/312-612 TRPV5-like Myripristis murdjan FISH-13 \| \| >XP_061145915.1/312-612 TRPV6 Syngnathus typhle FISH-14 \| \| >XP_027868757.1/312-612 TRPV5-like Xiphophorus couchianus FISH-15 \| \| >XP_062582294.1/394-699 TRPV5-like Saccostrea cucullata MOLLUSCA-1 \| \| >CAG2220307.1/388-698 TRPV5 Mytilus edulis MOLLUSCA-2 \| \| >XP_048774107.2/420-730 TRPV5-like Ostrea edulis MOLLUSCA-3 \| \| >XP_061164685.1/406-717 TRPV5-like Saccostrea echinata MOLLUSCA-4 \| \| >XP_011445409.2/418-731 TRPV5 Crassostrea gigas MOLLUSCA-5 \| \| >XP_025113281.1/401-707 TRPV5-like Pomacea canaliculata MOLLUSCA-6 \| \| >XP_021355545.1/407-714 TRPV5-like Mizuhopecten yessoensis MOLLUSCA-7 \| \| >XP_046366946.2/419-725 TRPV5-like Haliotis rufescens MOLLUSCA-8 \| \| >XP_033744456.1/373-651 TRPV5 Pecten maximus MOLLUSCA-9 \| \| >XP_052781744.1/377-654 TRPV5 Mya arenaria MOLLUSCA-10 \| \| >XP_060075724.1/373-651 TRPV5-like Ylistrum balloti MOLLUSCA-11 \| \| >XP_059150511.1/365-655 TRPV5-like Physella acuta MOLLUSCA-12 \| \| >XP_052231546.1/377-654 TRPV5-like Dreissena polymorpha MOLLUSCA-13 \| \| >XP_041349921.1/365-648 TRPV5-like Gigantopelta aegis MOLLUSCO-14 \| \| >XP_046544515.1/374-657 TRPV5-like Haliotis rubra MOLLUSCA-15 \| \| >CAG2197582.1/374-655 TRPV6 Mytilus edulis MOLLUSCA-16 \| \| >XP_029633752.1/376-656 TRPV5-like Octopus sinensis MOLLUSCA-17 \| \| >XP_046356888.2/374-657 TRPV6-like Haliotis rufescens MOLLUSCA-18 \| \| >XP_052822662.1/376-656 TRPV5 Octopus bimaculoides MOLLUSCA-19 \| \| >XP_050396983.1/369-663 TRPV6 Patella vulgata MOLLUSCA-20 \| \| >XP_021374011.1/232-510 TRPV6-like Mizuhopecten yessoensis MOLLUSCA-21 \| \| >GFR68752.1/391-699 TRPV6 Elysia marginata MOLLUSCA-22 \| \| >XP_053305982.1/376-675 TRPV5-like Spea bombifrons AMPHIBIA-1 \| \| >XP_063290780.1/290-589 TRPV5-like Pelobates fuscus AMPHIBIA-2 \| \| >NP_001186854.1/290-589 TRPV5 Xenopus tropicalis AMPHIBIA-3 \| \| >XP_053548766.1/308-607 TRPV5-like Bombina bombina AMPHIBIA-4 \| \| >XP_053305982.1/376-675 TRPV55-like Spea bombifrons AMPHIBIA-5 \| \| >XP_053547792.1/311-610 TRPV6-like Bombina bombina AMPHIBIA-6 \| \| >XP_053306450.1/311-610 TRPV6-like Spea bombifrons AMPHIBIA-7 \| \| >XP_040291407.1/312-611 TRPV6-like Bufo bufo AMPHIBIA-8 \| \| >XP_018080421.1/311-613 TRPV6 Xenopus laevis AMPHIBIA-9 \| \| >XP_018080408.1/290-589 TRPV5 Xenopus laevis AMPHIBIA-10 \| \| >XP_063291328.1/311-610 TRPV6-like Pelobates fuscus AMPHIBIA-11 \| \| >XP_040217657.1/311-609 TRPV6-like Rana temporaria AMPHIBIA-12 \| \| >XP_063819859.1/311-610 TRPV6 Pseudophryne corroboree AMPHIBIA-13 \| \| >XP_056385837.1/312-611 TRPV6 Hyla sarda AMPHIBIA-14 \| \| >XP_031762079.1/311-613 TRPV6 Xenopus tropicalis AMPHIBIA-15 \| \| >AFS63976.1/293-592 TRPV5/6 Xenopus laevis AMPHIBIA-16 \| |
| --- | --- | --- | --- | --- | --- | --- | --- | --- | --- | --- | --- | --- | --- | --- | --- | --- | --- | --- | --- | --- | --- | --- | --- | --- | --- | --- | --- | --- | --- | --- | --- | --- | --- | --- | --- | --- | --- | --- | --- | --- | --- | --- | --- | --- | --- | --- | --- | --- | --- | --- | --- | --- | --- | --- | --- | --- | --- | --- | --- | --- | --- | --- | --- | --- | --- | --- | --- | --- | --- | --- | --- | --- | --- | --- | --- | --- | --- | --- | --- | --- | --- | --- | --- | --- | --- | --- | --- | --- | --- | --- | --- | --- | --- | --- | --- | --- | --- | --- | --- | --- | --- | --- | --- | --- | --- | --- | --- | --- | --- | --- | --- | --- | --- | --- | --- | --- | --- | --- | --- | --- | --- | --- | --- | --- | --- | --- | --- | --- | --- | --- | --- | --- | --- | --- | --- | --- | --- | --- | --- | --- | --- | --- | --- | --- | --- | --- | --- | --- | --- | --- | --- | --- | --- | --- | --- | --- | --- | --- | --- | --- | --- | --- | --- | --- | --- | --- | --- | --- | --- | --- | --- | --- | --- | --- | --- | --- | --- |
