# Supplementary figures and images for: A novel TRPV5/6-like channel from a scleractinian coral
Source: PLoS One. 2025 Oct 7;20(10):e0332671. doi: 10.1371/journal.pone.0332671 (PMC12503276; doi:10.1371/journal.pone.0332671)

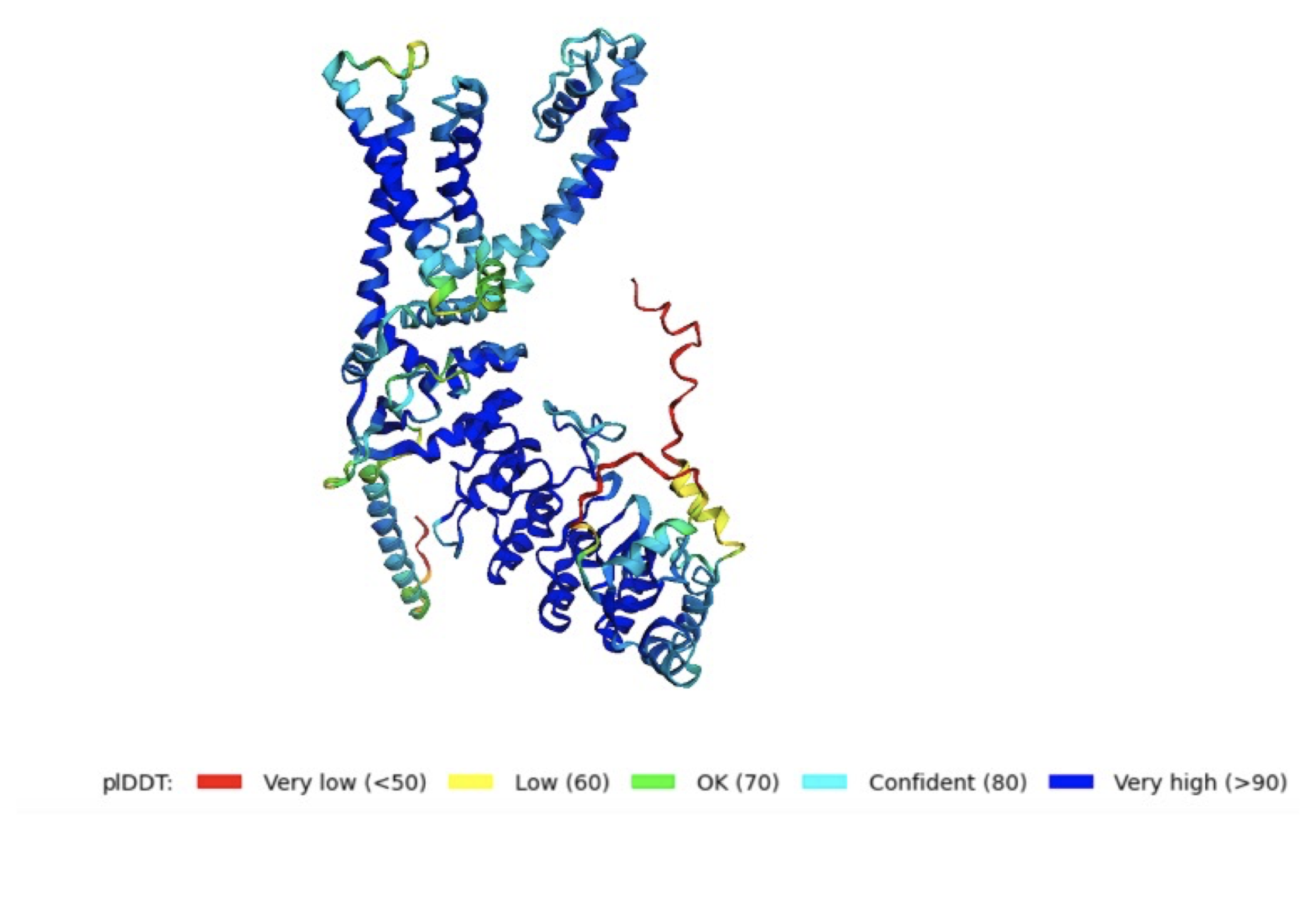

Supplement: S1 Fig — Alphafold model colored by the predicted local distance difference test score (pLDDT). A pLDDT ≥ 90 have very high model confidence, residues with 90 > pLDDT ≥ 70 are classified as confident, while residues with 70 > pLDDT > 50 have low confidence. Overall structure scores are provided in the table. Interface pTM scores provide the confidence score for the complete model (scored 0–1). (TIF) [file pone.0332671.s002.tif]

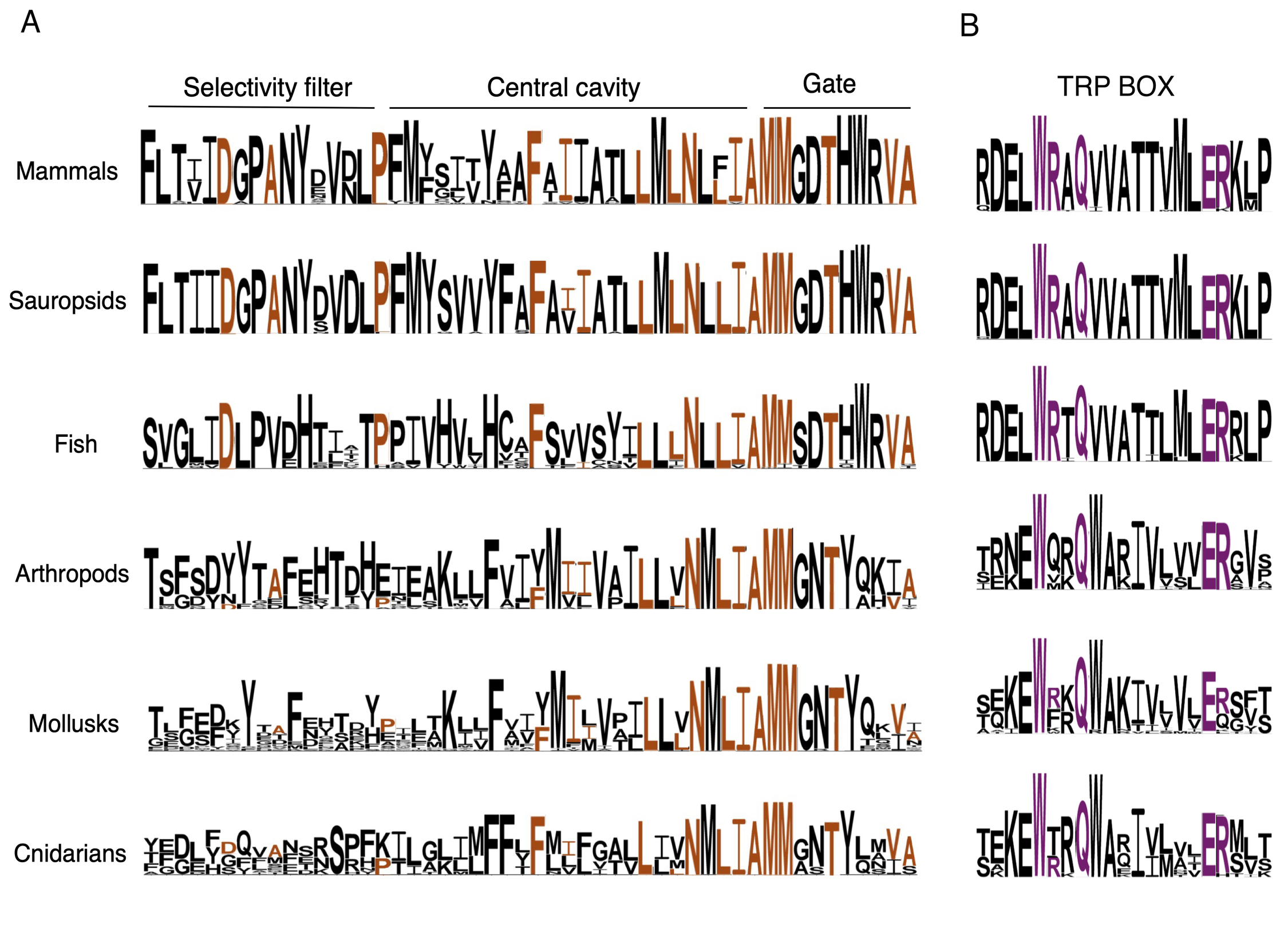

Supplement: S2 Fig — (A) Consensus amino acid sequences of the pore domain (selectivity filter, central cavity, and gate) for the indicated groups of organisms. The colored residues are the most conserved or demonstrated to have important functional roles in channel behavior. (B) Consensus amino acid sequences for the TRP box domain depicting highly conserved residues across mammals, sauropsids, fish, arthropods, mollusks, and cnidarians. (TIF) [file pone.0332671.s003.tif]

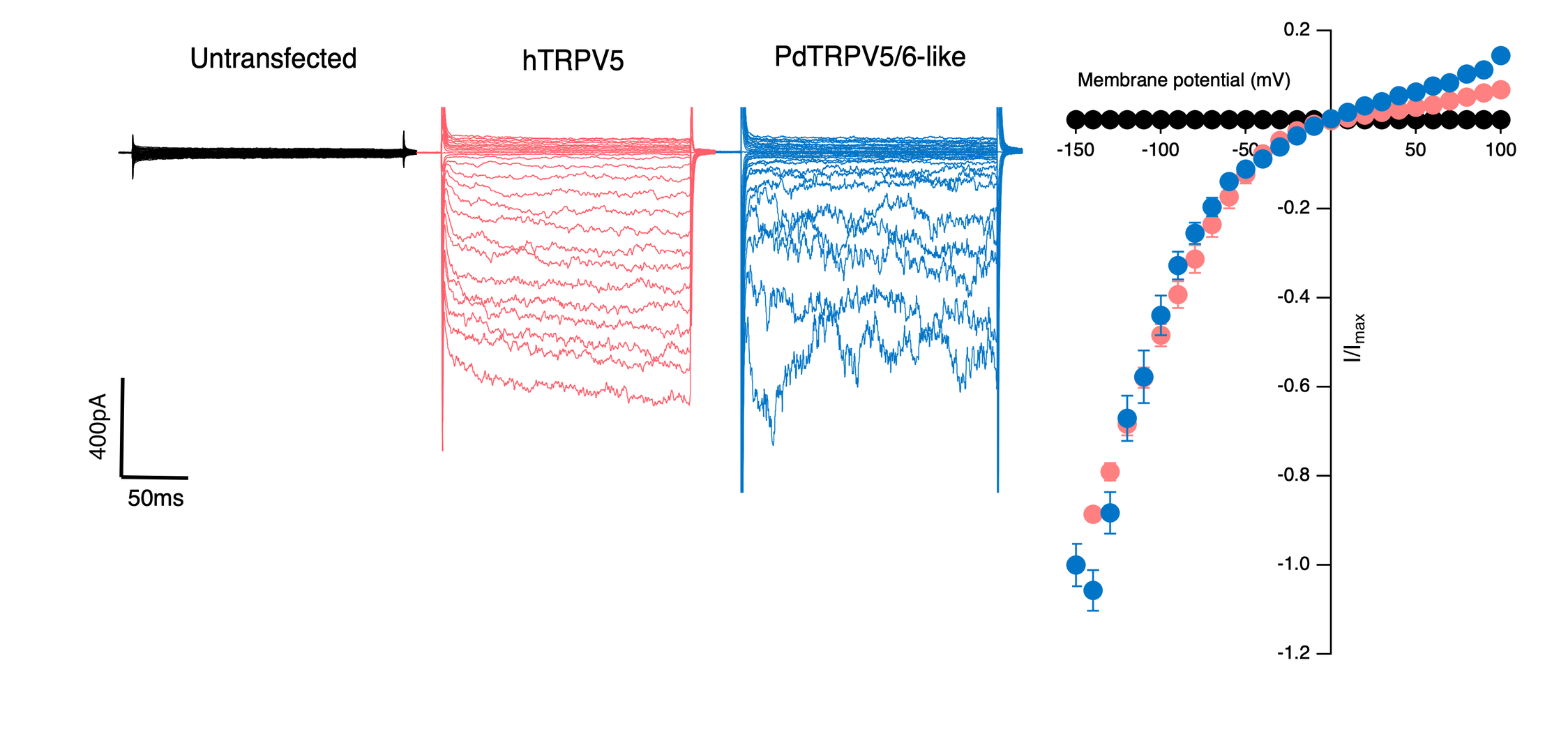

Supplement: S3 Fig — (A) Representative whole-cell current traces from untransfected HEK293 cells and cells transfected with either human TRPV5 or coral PdTRPV5/6-like channels. (B) The corresponding whole-cell current–voltage (I–V) relationships are shown, with error bars representing the standard error of the mean (± s.e.m.). (TIF) [file pone.0332671.s004.tif]

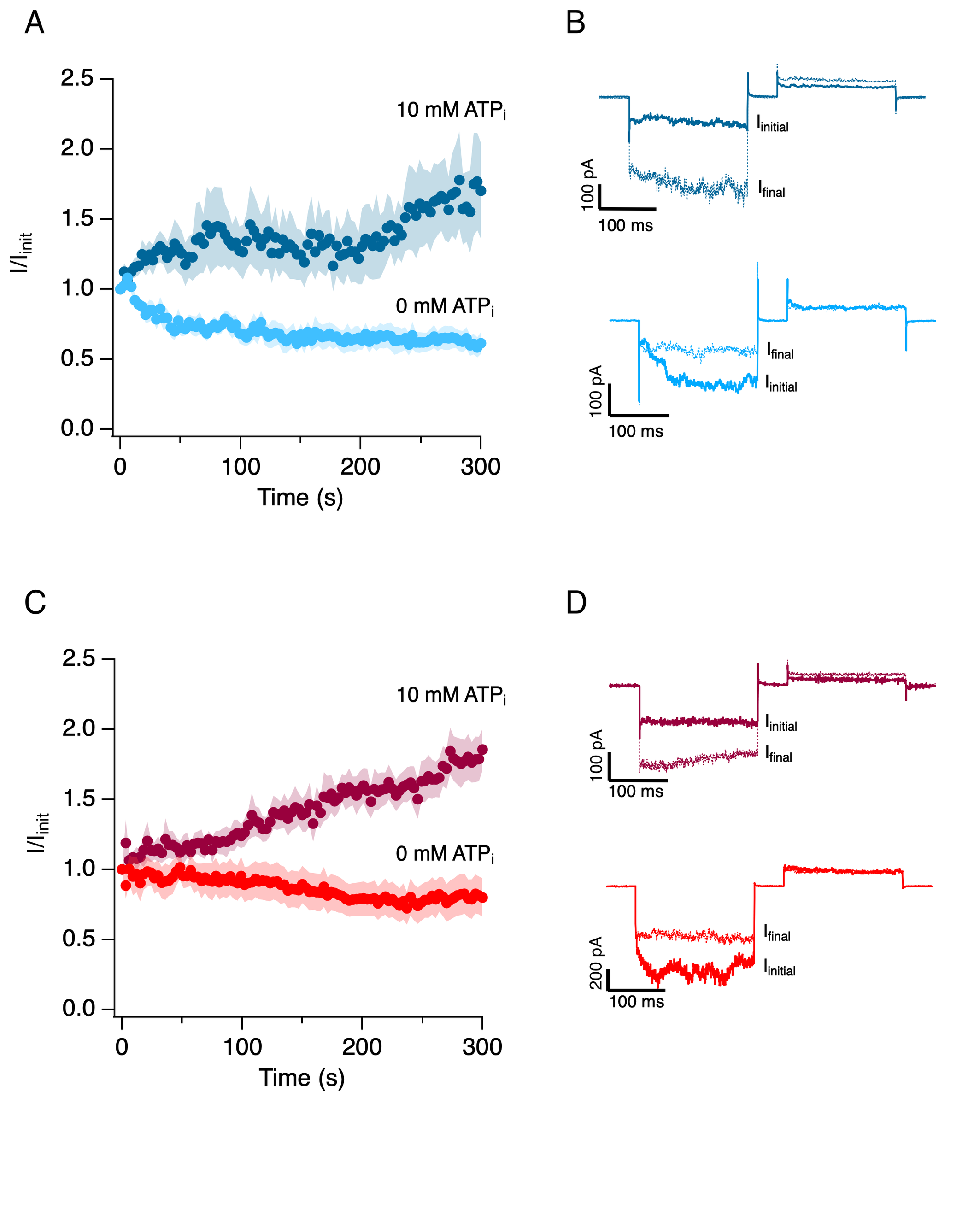

Supplement: S4 Fig — (A and C) Time course (5-min with 3s intervals) of monovalent currents recorded at −100 mV after patch excision in the outside-out configuration (light blue and orange circles) in the absence of ATP. The inward currents increased over time in the presence of 10mM ATP-diNa (n = 5) (dark blue and red circles) for both species, respectively. (B and D) Representative current traces with prolonged exposure to ATP-diNa, which prevents rundown of TRPV5 channels in symmetrical conditions of monovalent cations. The dotted line indicates the final current after 5 minutes. Normalized currents show the average of each experiment with error bars for both conditions (shadows). Data are mean ± s.e.m. (TIF) [file pone.0332671.s005.tif]
